# Supplementary material for: Highly efficient electrosynthesis of hydrogen peroxide on a superhydrophobic three-phase interface by natural air diffusion
Source: Nat Commun. 2020 Apr 7;11:1731. doi: 10.1038/s41467-020-15597-y (PMC7138826; doi:10.1038/s41467-020-15597-y)
Supplement: Supplementary file 1 — Supplementary Information [file 41467_2020_15597_MOESM1_ESM.pdf]

Supplementary Information for

Highly efficient electrosynthesis of hydrogen peroxide on a superhydrophobic three-phase interface by natural air diffusion

Zhang *et al.*

Supplementary Figures

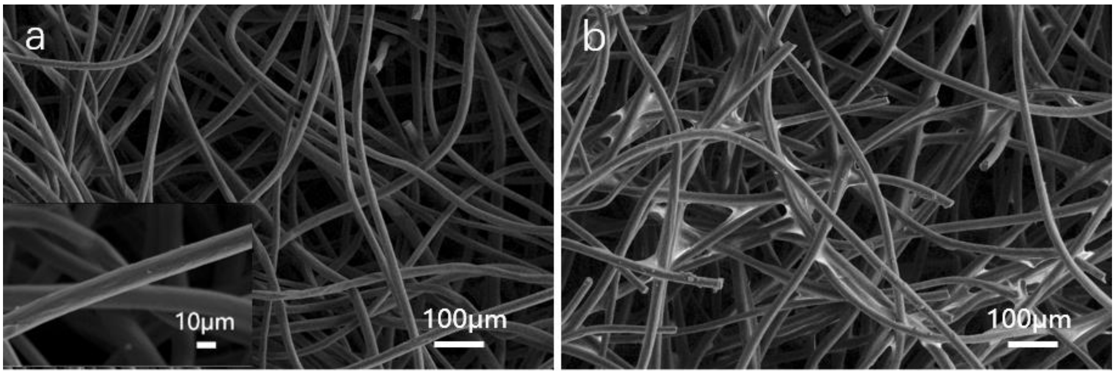

Supplementary Figure 1. SEM images of CF, with insert shows fibers (a) before and (b) after modification with PTFE suspension.

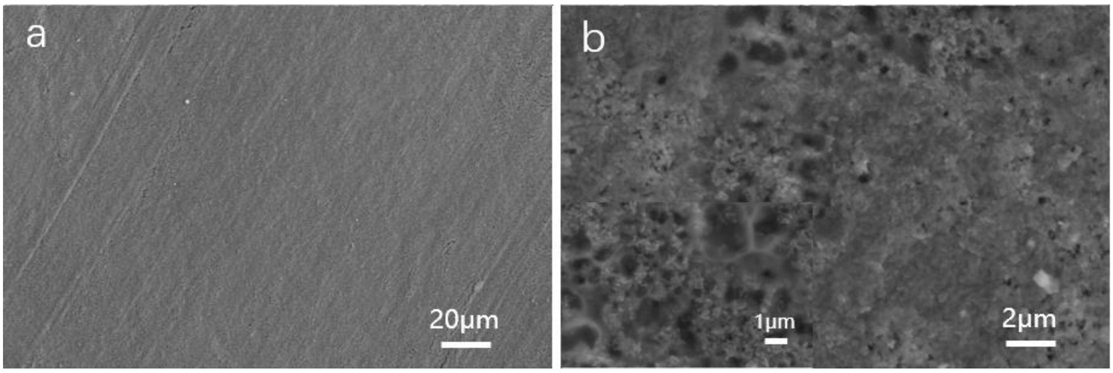

Supplementary Figure 2. SEM images of (a) traditional GDE diffusion layer and (b) magnified view of the tunnel.

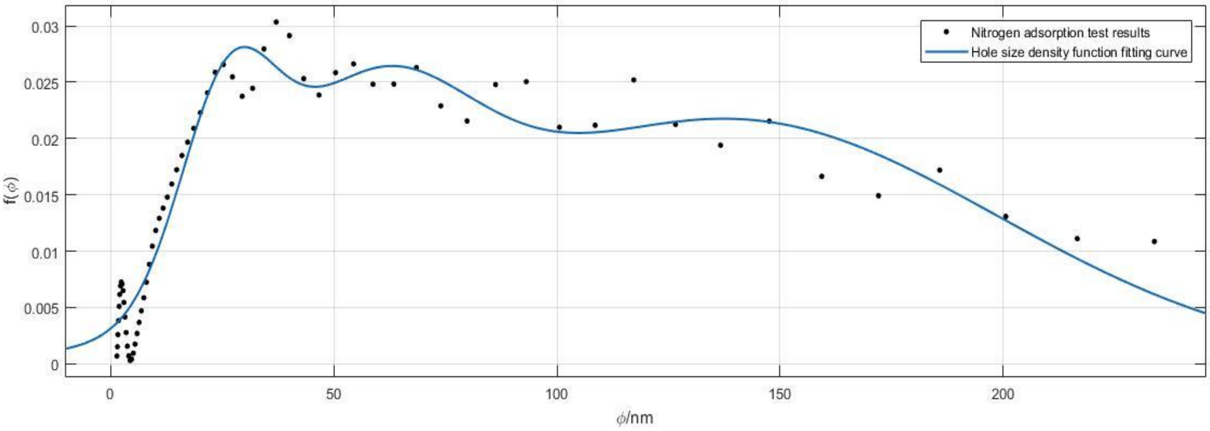

Supplementary Figure 3. Diffusion layer pore size distribution of traditional GDE.

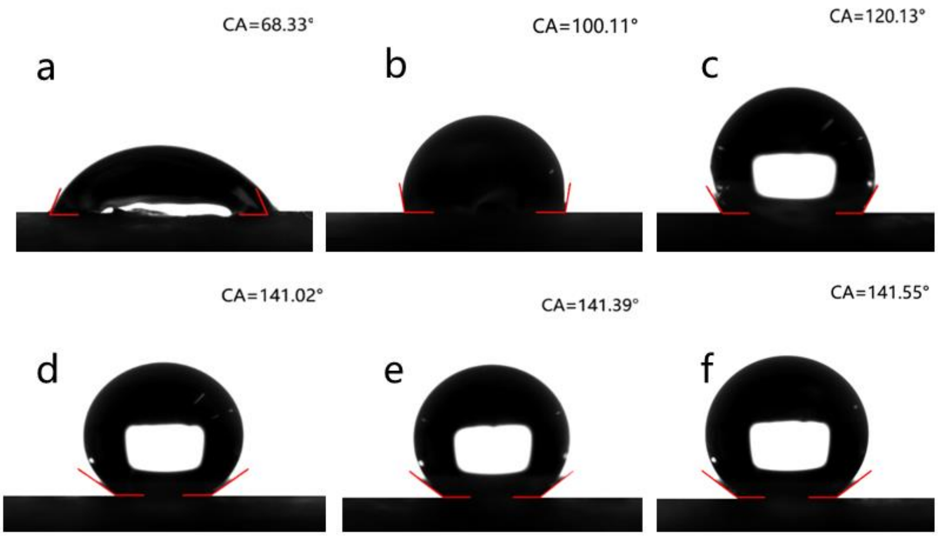

Supplementary Figure 4. Photographs of contact angle of catalytic layers with (a) PTFE / CB =0.1; (b) PTFE / CB =0.2; (c) PTFE / CB =0.3; (d) PTFE / CB =0.6; (e) PTFE / CB =1; (f) PTFE / CB =1.5.

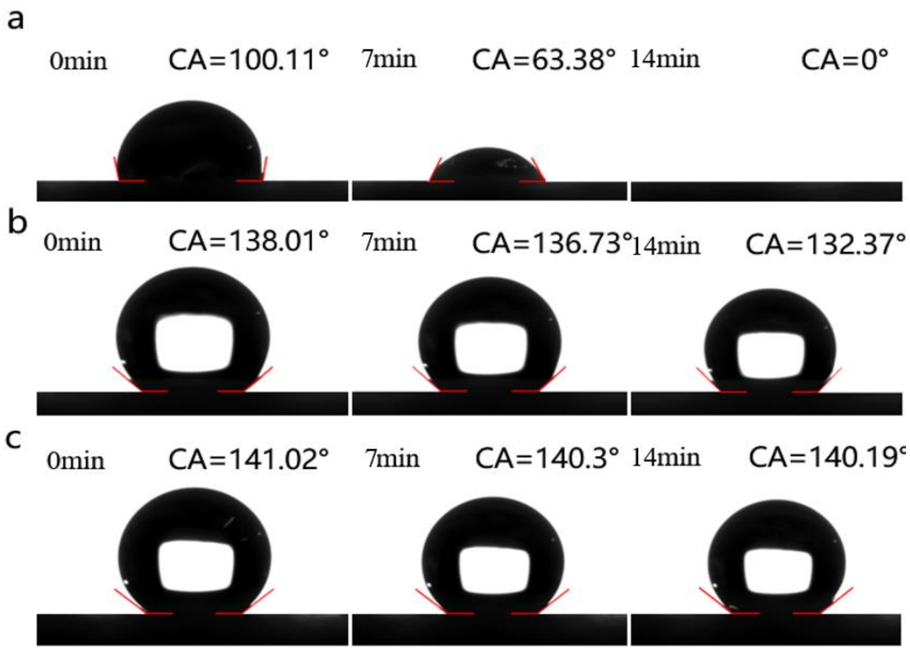

Supplementary Figure 5. Photographs of the contact angle of the catalytic layers as a function of time with (a) PTFE / CB =0.2; (b) PTFE / CB =0.5; (c) PTFE / CB =0.6.

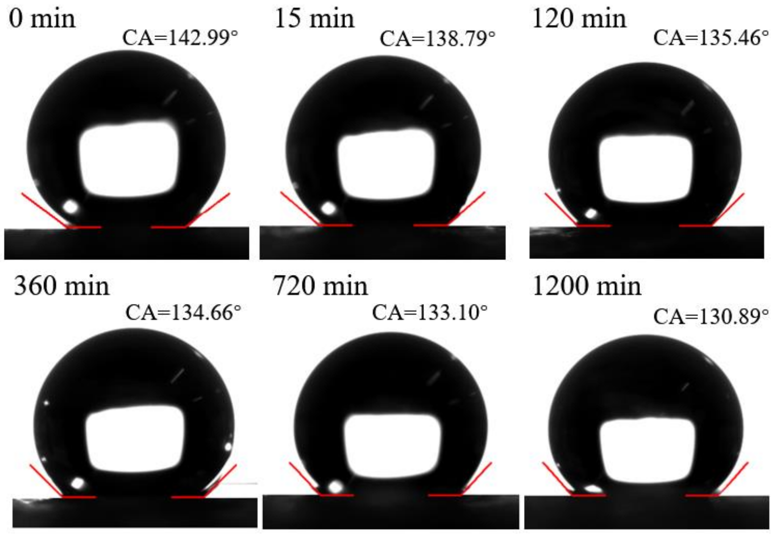

Supplementary Figure 6. Photographs of the contact angle of the catalytic layer (PTFE / CB =0.6) as a function of electrolysis time at the applied current density of 60 mA cm^{-2}.

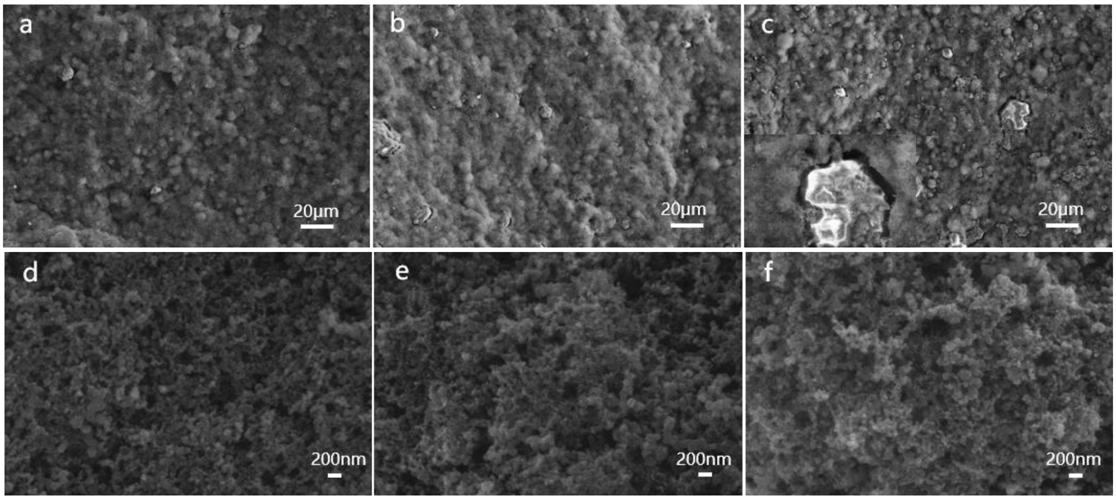

Supplementary Figure 7. SEM images of catalytic layers with (a) PTFE / CB =0.1; (b) PTFE / CB =0.6 and (c) PTFE / CB =1.5 at low magnification; SEM images of PTFE/carbon black mixture with (d) PTFE / CB =0.1; (e) PTFE / CB =0.6 and (f) PTFE / CB =1.5 at high magnification.

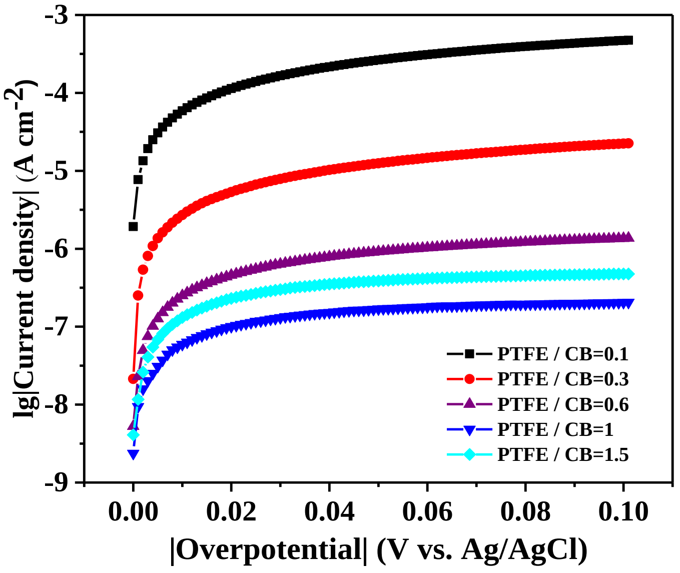

Supplementary Figure 8. Tafel plot of catalytic layer materials with different PTFE/CB mass ratio.

Source data are provided as a Source Data file.

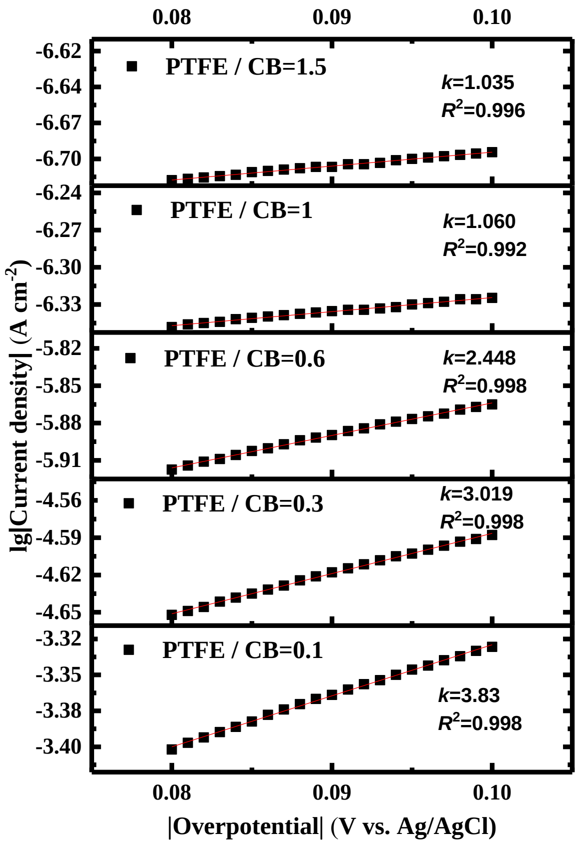

Supplementary Figure 9. The linear fit for the Tafel plots of the overpotential from 80 to 100 mV.

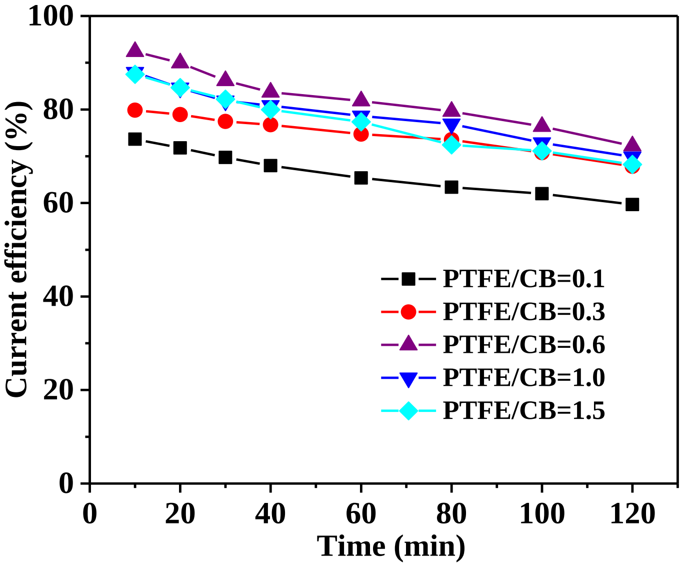

Supplementary Figure 10. Current efficiency of different PTFE/CB NADEs as function of electrolysis time at the current density of 60 mA cm^{-2}. Source data are provided as a Source Data file.

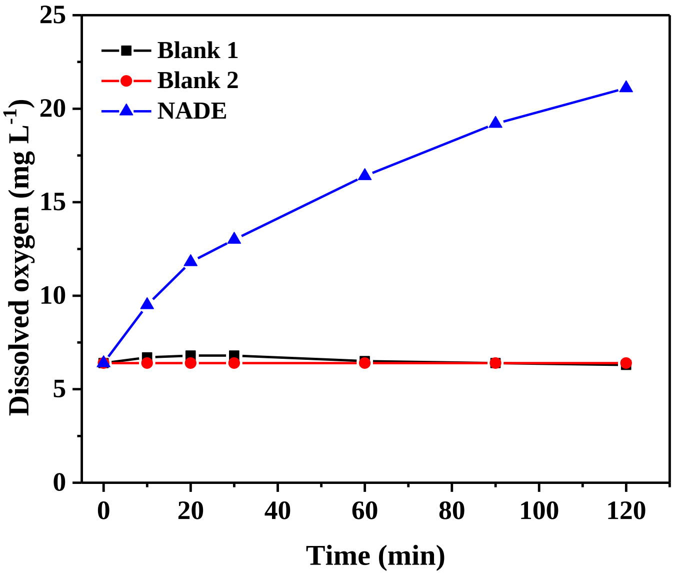

Supplementary Figure 11. Change of dissolved oxygen concentration with electrolysis time in different systems. Blank 1 was the system shown in Fig. 4a-C with almost no generation of H_2O_2 which measured the contribution of oxygen produced by the anodic oxidation to the change in DO; Blank 2 was NADE system without voltage applied which measured the contribution of naturally diffused oxygen to the change in DO. Source data are provided as a Source Data file.

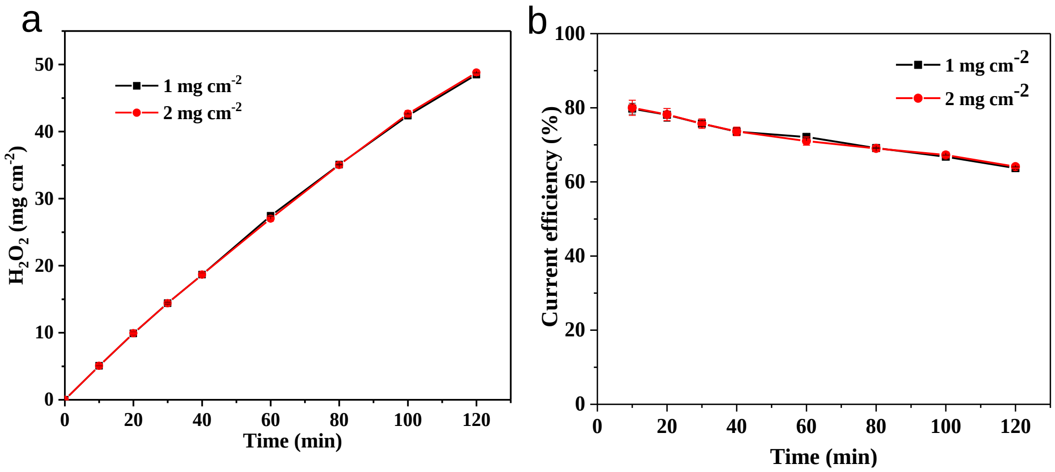

Supplementary Figure 12. (a) The production of H_2O_2 and (b) current efficiency under the current density of 60 mA cm^{-2} with different loadings of catalyst. Source data are provided as a Source Data file.

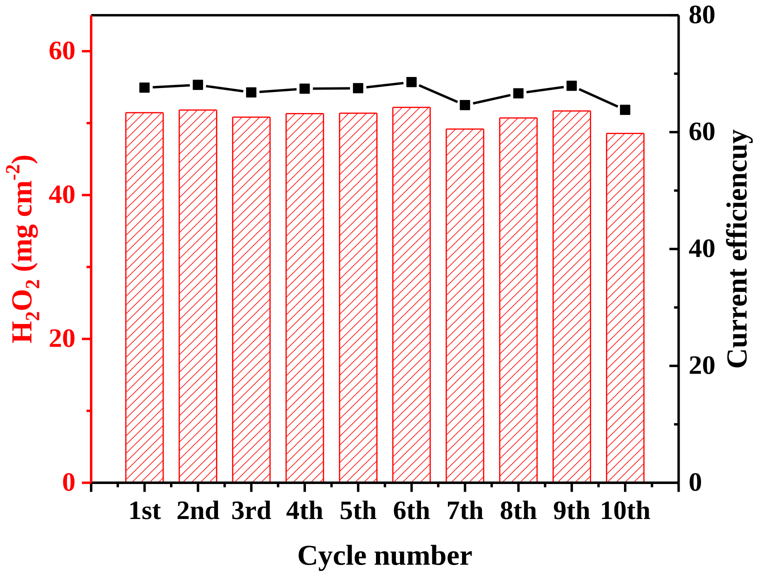

Supplementary Figure 13. The stability test of the NADE with the catalyst loading of 1 mg cm^{-2} in 10-times (20 h) continuous runs. Source data are provided as a Source Data file.

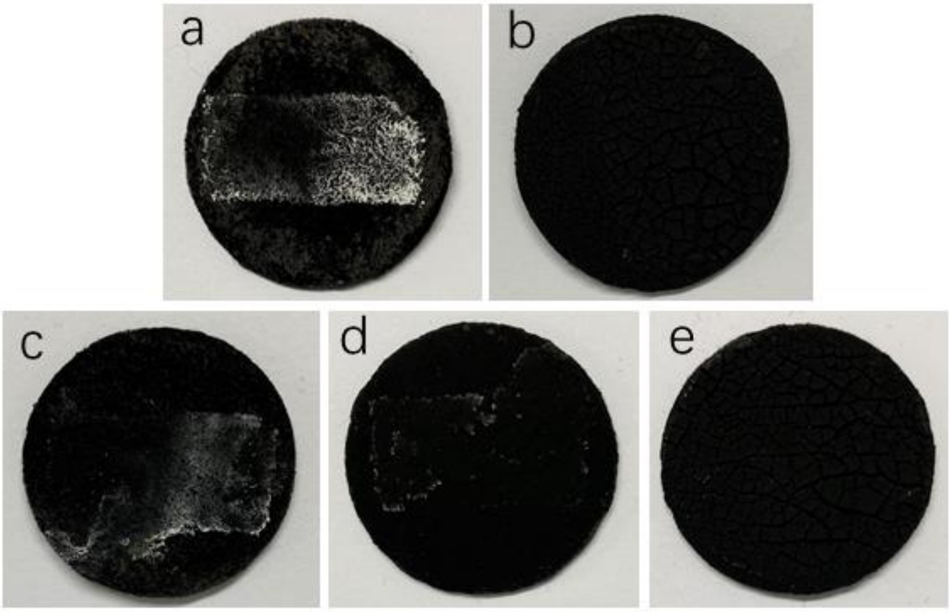

Supplementary Figure 14. The Photographs of used NADEs with different catalyst loadings. (a) NADE with catalyst loading of 1 mg cm^{-2} after running 10 times (20 h) at the current density of 60 mA cm^{-2}; (b) NADE with catalyst loading of 13.2 mg cm^{-2} after running 10 times (20 h) at the current density of 60 mA cm^{-2}; NADE with catalyst loadings of (c) 2 mg cm^{-2}; (d) 4.4 mg cm^{-2}; (e) 8.8 mg cm^{-2} after running 2 h at the current density of 200 mA cm^{-2}.

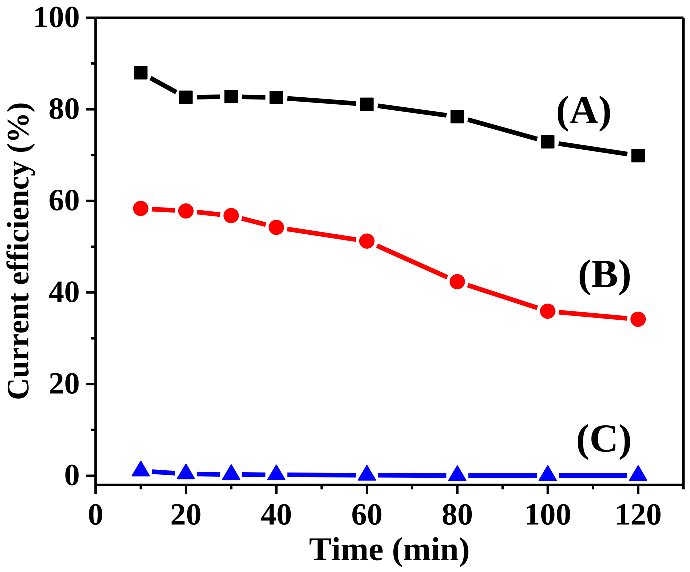

Supplementary Figure 15. The current efficiency of H_2O_2 production by different systems under 60 mA cm^{-2} current density. Source data are provided as a Source Data file.

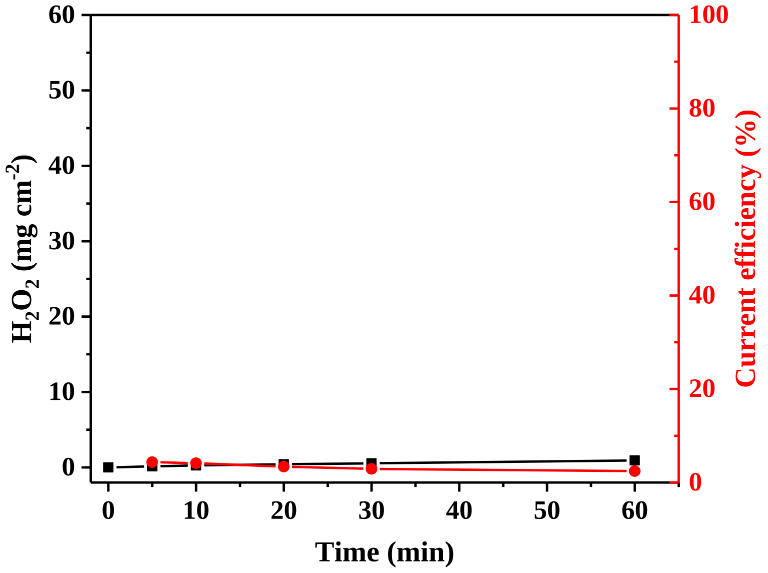

Supplementary Figure 16. The production of H_2O_2 and current efficiency of the carbon felt substrate without loading catalytic layer under the current density of 60 mA cm^{-2}. Source data are provided as a Source Data file.

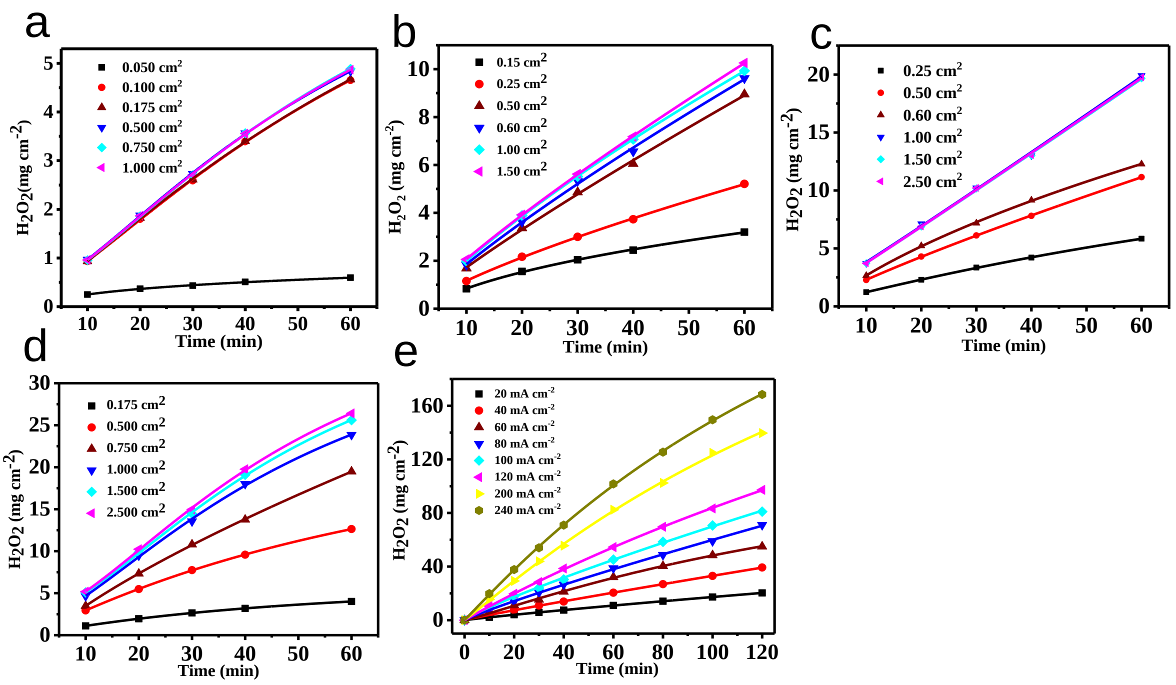

Supplementary Figure 17. The production of H_2O_2 with different oxygen diffusion areas under the current density of (a) 10 mA cm^{-2}, (b) 20 mA cm^{-2}, (c) 40 mA cm^{-2} and (d) 60 mA cm^{-2}; (e) the H_2O_2 production of NADE under different current densities. Source data are provided as a Source Data file.

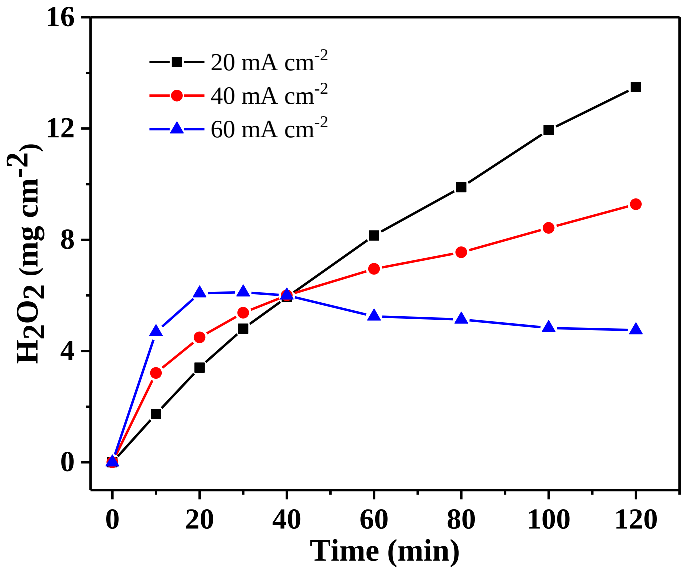

Supplementary Figure 18. H_2O_2 concentration produced as a function of time for normal GDE without pumping air at constant current. Source data are provided as a Source Data file.

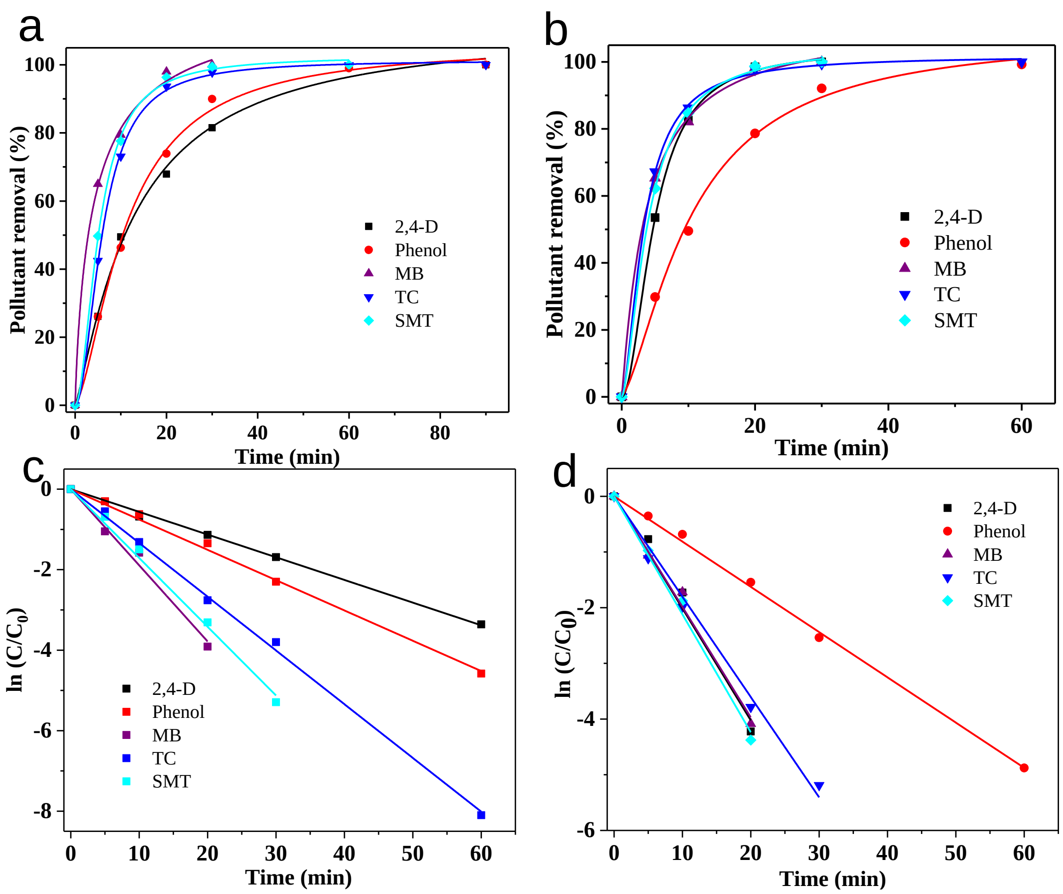

Supplementary Figure 19. The different organic pollutants removal in (a); (c) EF and (b); (d) PEF processes with the NADE. Source data are provided as a Source Data file.

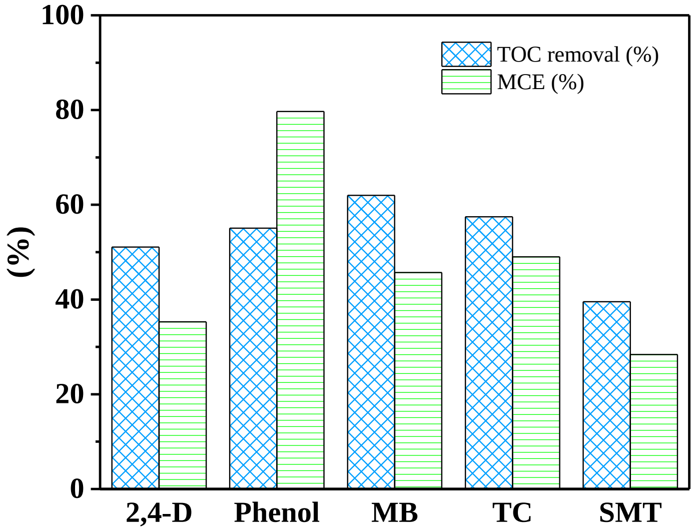

Supplementary Figure 20. TOC removal and MCE of different pollutants in EF process with the NADE; current density of 20 mA cm^{-2}, 0.5 mM Fe^{2+} as catalyst. Source data are provided as a Source Data file.

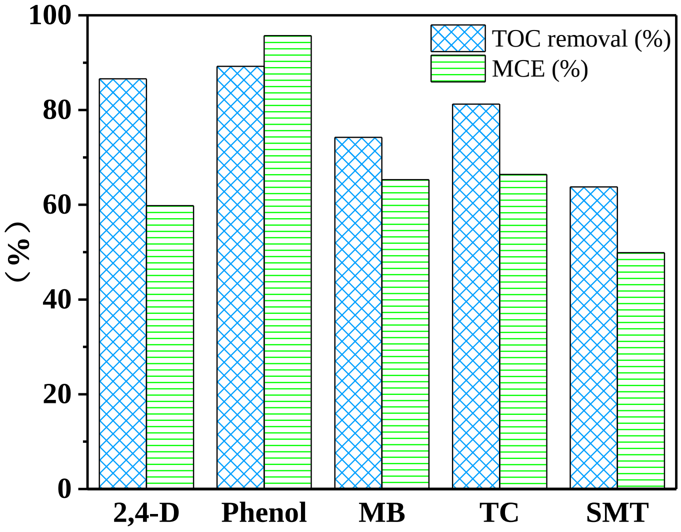

Supplementary Figure 21. TOC removal and MCE of different pollutants in PEF process with NADE; current density of 20 mA cm^{-2}, 0.5 mM Fe^{2+} as catalyst. Source data are provided as a Source Data file.

Supplementary Tables

Supplementary Table 1. BET surface area (S_{BET}), micropore area (S_{micro}), total pore volume (V_{tol}), and pore size of catalytic layer materials with different PTFE/CB mass ratio.

	0.1	0.3	0.6	1.0	1.5
S_{BET} (m^2 g^{-1})	171.34	144.68	95.41	66.30	43.85
S_{micro} (m^2 g^{-1})	61.14	42.46	9.10	4.97	4.46
V_{tol} (cm^3 g^{-1})	0.37	0.34	0.30	0.23	0.17
V_{micro} (cm^3 g^{-1})	0.03	0.02	0.003	0.0007	0.0004
Pore size (nm)	15.62	15.82	18.06	18.56	19.50

Supplementary Table 2. Linear fit equations and exchange current densities calculated from the linear region of the Tafel plots.

PTFE/CB ratio	Fit linear equation	$10^{-6} j_0$ (A cm^{-2})
1.5	y=-6.79547+1.035x	0.16
1	y=-6.42836+4.74288x	0.39
0.6	y=-6.10879+4.74288x	0.79
0.4	y=-4.89066+4.74288x	12.9
0.1	y=-3.70859+4.74288x	194.9

Supplementary Table 3. The comparison with the performance of H_2O_2 production under different current densities.

Current density (mA cm^{-2})	H_2O_2 generation rate (mg h^{-1} cm^{-2})	CE (%)	EEC (kWh kg_{H2O2}^{-1})	References
20	10.97	86.5	4.6	This work
60	32.09	81.8	6.9	This work
120	54.53	71.2	12.9	This work
200	84.37	66.9	18.9	This work
240	100.67	64.8	19.4	This work
107	10.7	17	13.2	[1]
217	38.33	28	11.3	[1]
275	40	23	20.4	[1]
145	26.69	29	-	[2]

Supplementary Table 4. The comparison with the performance of H_2O_2 production in literatures.

Cathode	Experimental conditions	Oxygen pumping volume	H_2O_2 generation rate (mg h^{-1} cm^{-2})	EEC (kWh kg_{H2O2}^{-1})	Energy consumption of Aeration (kWh kg_{H2O2}^{-1})	OE (%)	CE (%)	References
GDE	Cathode area 6.15 cm^2, V 250 mL, pH 3, 0.05 M Na_2SO_4, I 30 mA cm^{-2}	1.2 L min^{-1} (air)	12.85	–	–	0.37	50	[3]
GDE	Cathode area 100 cm^2, V 25 L, pH 7, 0.05 M Na_2SO_4, I 65 mA cm^{-2}	5 L min^{-1} (air)	13.93	53.86	1077	1.59	33.8	[4]
GDE	Cathode area 3.4 cm^2, V 400 mL, pH 2, 0.1 M K_2SO_4, E = -1.1 V vs. SCE	0.2 bar (O_2)	1.12	18.8	–	–	–	[5]
GDE	Cathode area 20 cm^2, V 1.5 L, pH 1, 0.1 M K_2SO_4 & 0.1 M H_2SO_4, E = -2.25 V vs. SCE	–	15.52	22.1	–	–	–	[6]
GDE	Cathode area 3.1 cm^2, V 100 mL, pH 3, 0.05 M Na_2SO_4, I 145 mA cm^{-2}	2 L min^{-1} (O_2)	26.69	–	–	4.90	29	[2]
GDE	Cathode area 4 cm^2, V 200 mL, pH 3, 0.05 M Na_2SO_4, I 20 mA cm^{-2}	2 L min^{-1} (air)	11.78	–	–	0.13	93	[7]
GDE	Cathode area 7×2 cm^2, V 200 mL, pH 7, 0.05 M Na_2SO_4, I 35.7 mA cm^{-2}	0.5 L min^{-1} (air)	12.16	15.9	58.8	1.93	51–88	[8]
GDE	Cathode area 7×2 cm^2, V 200 mL, pH 7, 0.05 M Na_2SO_4, I 35.7 mA cm^{-2}	0.1 L min^{-1} (air)	11.16	17.9	64.1	8.84	45–84	[8]
NADE	Cathode area 5 cm^2, V 250 mL, pH 7, 0.05 M Na_2SO_4, I 20 mA cm^{-2}	0	10.97	4.55	0	44.5–64.9	86.5	Present work
NADE	Cathode area 5 cm^2, V 250 mL, pH 7, 0.05 M Na_2SO_4, I 60 mA cm^{-2}	0	30.93	6.92	0	44.5–64.9	81.82	Present work
NADE	Cathode area 5 cm^2, V 250 mL, pH 7, 0.05 M Na_2SO_4, I 240 mA cm^{-2}	0	101.67	19.35	0	44.5–64.9	66.79	Present work

Supplementary Table 5. The comparison of energy consumption of TOC removal with literatures.

Method	Electrode (anode/cathode)	Experimental conditions	Pollutant degradation	TOC removal	EEC (kWh $kgTOC^{-1}$)	References
PEF	Pt/GDE	350 mL of 100 mg L^{-1} phenol, 0.1 M H_2SO_4 and 0.1 M K_2SO_4, 3 mM Fe^{2+}, 0.2 bar (O_2)	65%, 2 h	11%	-	[9]
PEF	Graphite/MesoC /GF	70 mL of 50 mg L^{-1} phenol, 0.05 M Na_2SO_4, pH=3, 50 mL min^{-1} (O_2)	100%, 3 h	85% (COD)	-	[10]
SPEF	BDD/GDE	2.5L of 0.28 mM bronopol, 0.05 M Na_2SO_4, pH=3, 0.5 mM Fe^{2+}, 8.6 kPa (Air)	100%, 6 h	94%	3900	[11]
EF	Pt/Gr-Brush	400 mL of 0.33 mM phenol, 0.05 M K_2SO_4, pH=3, 0.1 mM Fe^{2+}, 0.2 L min^{-1} (Air)	99%, 2 h	40%,	100	[12]
SPEF	BDD/GDE	75 L of 50 mg L^{-1} pyrimethanil and 90 mg L^{-1} methomyl, 0.05 M Na_2SO_4, pH=3, 0.5 mM Fe^{2+}, 10 L min^{-1} (Air)	70-77%, 2 h	15%	938	[4]
EF	Pt/GDE	2.5 L of 92 mg L^{-1} 2,4-D, 0.05 M Na_2SO_4, pH=3, 0.5 mM Fe^{2+}, 8.6 kPa (Air)	-	59%, 5 h	420	[13]
EF	BDD/GDE	350mL of 91.23 mg L^{-1} Orange II, 0.1 M H_2SO_4 and 0.1 M K_2SO_4, pH=3, 0.5 mM Fe^{2+}, 0.2 bar (O_2)	69%, 2 h	-	-	[14]
EF	DSA/Fe@PTFE /MWCNT-MF	100 mL of 10.0 mg L^{-1} MB, 0.05 M Na_2SO_4, pH=3, 0.5 L min^{-1} (Air)	100%, 1 h	78.4%	-	[15]
EF	$Ti_4O_7/Fe^{II}Fe^{III}$ LDH/CF	145 mL of 50.7 mg L^{-1} SMT, 0.05 M Na_2SO_4, pH=3, 1 L min^{-1} (Air)	100%, 40 min	97%, 8 h	-	[16]
EF	BDD/CF	230 mL of 89 mg L^{-1} TC, 0.05 M Na_2SO_4, pH=5.94, 1.0 g L^{-1} chalcopyrite, 1 L min^{-1} (Air)	100%, 10 min	98%, 6 h	-	[17]
EF	DSA/NADE	250 mL of 100 mg L^{-1} 2,4-D, 0.05 M Na_2SO_4, pH=3, 0.5 mM Fe^{2+}	100%, 1.5 h	51.1%	66.4	This work
EF	DSA/NADE	250 mL of 100 mg L^{-1} phenol, 0.05 M Na_2SO_4, pH=3, 0.5 mM Fe^{2+}	100%, 1.5 h	55.1%	36.6	This work
EF	DSA/NADE	250 mL of 100 mg L^{-1} MB, 0.05 M Na_2SO_4, pH=3, 0.5 mM Fe^{2+}	100%, 1 h	62%	65.8	This work
EF	DSA/NADE	250 mL of 100 mg L^{-1} SMT, 0.05 M Na_2SO_4, pH=3, 0.5 mM Fe^{2+}	100%, 1 h	39.5%	91.7	This work
EF	DSA/NADE	250 mL of 100 mg L^{-1} TC, 0.05 M Na_2SO_4, pH=3, 0.5 mM Fe^{2+}	100%, 1 h	57.5%	52.7	This work

Supplementary References

1. Barros, W. R. P., Ereno, T., Tavares, A. C. & Lanza, M. R. V. In situ electrochemical generation of hydrogen peroxide in alkaline aqueous solution by using an unmodified gas diffusion electrode. *ChemElectroChem* **2**, 714-719 (2015).
2. Brillas, E., Calpe, J. C. & Casado, J. Mineralization of 2,4-D by advanced electrochemical oxidation processes. *Water Res.* **34**, 2253-2262 (2000).
3. Panizza, M. & Cerisola, G. Electrochemical generation of H_2O_2 in low ionic strength media on gas diffusion cathode fed with air. *Electrochim. Acta* **54**, 876-878 (2008).
4. Salmeron, I. et al. Optimization of electrocatalytic H_2O_2 production at pilot plant scale for solar-assisted water treatment. *Appl. Catal. B* **242**, 327-336 (2019).
5. Carneiro, J. F., Rocha, R. S., Hammer, P., Bertazzoli, R. & Lanza, M. R. V. Hydrogen peroxide electrogeneration in gas diffusion electrode nanostructured with Ta_2O_5. *Appl. Catal. A* **517**, 161-167 (2016).
6. Reis, R. M. et al. Use of gas diffusion electrode for the in situ generation of hydrogen peroxide in an electrochemical flow-by reactor. *Ind. Eng. Chem. Res.* **51**, 649-654 (2012).
7. Sheng, Y. et al. Electrogeneration of hydrogen peroxide on a novel highly effective acetylene black-PTFE cathode with PTFE film. *Electrochim Acta* **56**, 8651-8656 (2011).
8. Yu, X., Zhou, M., Ren, G. & Ma, L. A novel dual gas diffusion electrodes system for efficient hydrogen peroxide generation used in electro-Fenton. *Chem. Eng. J.* **263**, 92-100 (2015).
9. Assumpcao, M. H. M.T. et al. Low tungsten content of nanostructured material supported on carbon for the degradation of phenol. *Appl. Catal. B* **142**, 479-486 (2013).
10. Yue, D. et al. A metal-free visible light active photo-electro-Fenton-like cell for organic pollutants degradation. *Appl. Catal. B* **229**, 211-217 (2018).
11. Ye, Z., Guelfi, D. R. V., Alvarez, G., Alcaide, F., Brillas, E. & Sires, I. Enhanced electrocatalytic production of H_2O_2 at Co-based air-diffusion cathodes for the photoelectro-Fenton treatment of bronopol. *Appl. Catal. B* **247**, 191-199 (2019).
12. Mousset, E., Wang, Z., Hammaker, J. & Lefebvre, O. Electrocatalytic phenol degradation by a novel nanostructured carbon fiber brush cathode coated with graphene ink. *Electrochim. Acta* **258**, 607-617 (2017).
13. Garcia, O., Isarain-Chavez, E., El-Ghenymy, A., Brillas, E. & Peralta-Hernandez, J. M. Degradation of 2,4-D herbicide in a recirculation flow plant with a Pt/air-diffusion and a BDD/BDD cell by electrochemical oxidation and electro-Fenton process. *J. Electroanal. Chem.* **728**, 1-9 (2014).
14. Paz, E. C. et al. Evaluation of H_2O_2 electrogeneration and decolorization of Orange II azo dye using tungsten oxide nanoparticle-modified carbon. *Appl. Catal. B* **232**, 436-445 (2018).
15. Zhang, Y. et al. Design of a novel and efficient electro-Fenton cathode with skeleton of melamine foams. *Mater. Lett.* **239**, 196-199 (2019).
16. Ganiyu, S. O. et al. Electrochemical mineralization of sulfamethoxazole over wide pH range using (FeFeIII)-Fe-II LDH modified carbon felt cathode: Degradation pathway, toxicity and reusability of the modified cathode. *Chem. Eng. J.* **350**, 844-855 (2018).
17. Barhoumi, N. et al. Kinetics of oxidative degradation/mineralization pathways of the antibiotic tetracycline by the novel heterogeneous electro-Fenton process with solid catalyst chalcopyrite. *Appl. Catal. B* **209**, 637-647 (2017).
